# Prevalence of First Carpometacarpal Joint Osteoarthritis and Carpal Tunnel Syndrome Among Dentists in Saudi Arabia

**DOI:** 10.7759/cureus.23876

**Published:** 2022-04-06

**Authors:** Haifa AlKhodier, Mohammad Alqahtani, Abdulaziz Alshenaifi, Mazen Alnuwaiser

**Affiliations:** 1 Dentistry: Pediatric, King Faisal Specialist Hospital & Research Centre, Riyadh, SAU; 2 General Dentistry, King Faisal Specialist Hospital & Research Centre, Riyadh, SAU; 3 Plastic Surgery, Ministry of National Guard - Health Affairs, Riyadh, SAU; 4 Dentistry, Prince Abdulrahman Advanced Dental Institute, Riyadh, SAU

**Keywords:** dentists, carpometacarpal joint, hand pain, dentistry, carpal tunnel syndrome

## Abstract

Objectives

This study aims to determine the prevalence of the first carpometacarpal (CMC) joint osteoarthritis and Carpal Tunnel Syndrome (CTS) among dentists from different specialties in Saudi Arabia and their association with gender, years of practice, and weekly working hours.

Materials and Methods

In this cross-sectional study, 361 dentists in Saudi Arabia have completed an online questionnaire of three parts: demographic and health data, the Thumb Disability Exam (TDX), and the Boston Carpal Tunnel Syndrome Questionnaire (BCTQ). Univariate and multivariate analyses of logistic regression were performed to investigate the participants' predictors of the first CMC joint osteoarthritis and Carpal Tunnel Syndrome. The level of significance was set at α = 0.05 for all tests.

Results

Thumb disability was significantly associated with the female gender (aOR 2.21; 95 percent CI 1.31-3.56) and dentists aged 50 or older (aOR 9.63; 95 percent CI 1.05-88.47). The symptom severity scale (SSS) part of BCTQ was significantly associated with increased risk in the female gender (aOR 1.62; 95% CI 1.62-2.58). Limiting the clinical work to 10-20 hours per week showed a significant reduction in the odds of reporting CTS symptoms in SSS (aOR 0.44; 95% CI 0.21-0.90). CTS-related hand disability was more likely to be reported by the female gender (aOR 2.21; 95% CI 1.36-3.57) and less likely to be reported by endodontic specialists (aOR 0.15; 95% CI 0.04-0.58).

Conclusion

The female gender was significantly associated with first CMC joint osteoarthritis and CTS among dentists in Saudi Arabia. Other predictors were also identified in this cross-sectional study.

## Introduction

Musculoskeletal disorders are one of the leading causes of chronic pain, disability, and decreased productivity among employees. They are considered a significant cause of absence in developed countries [1-3]. Some challenging professions, such as dentistry, require maintaining specific postures over a long period [4]. Many physiological micro-changes may affect the muscles, tendons, joints, ligaments, and bones, predisposing their biological structures to discomfort, pain, and eventually musculoskeletal disorders (MSDs) [5].

Due to associated continuous strains, back, neck, shoulder, and wrist pain are common among dentists. In addition, any improper posture increases the likelihood of developing these disorders [6-8]. Many studies have shown that females, in particular, are at higher risk of having MSDs [8-12]. In Saudi Arabia, MSDs among dentists were high in the Qassim and Ha'il region [13,14]. Similarly, MSDs among dental practitioners in Jeddah and Riyadh were 70% and 90.2%, respectively [11,12]. Moreover, many authors reported an increased association between the dental profession and hand/wrist pain [8,15-22].

This study aimed to determine the prevalence of the first CMC joint osteoarthritis and Carpal Tunnel Syndrome among dentists from different specialties in Saudi Arabia and their association with gender, age, years of practice, and weekly working hours.

## Materials and methods

Study design and sample size

This cross-sectional study recruited a group of 361 Saudi dentists who have recently updated their contact information through the Saudi Commission for Health Specialties registry and have been practicing dentistry for at least one year. Initially, 466 dentists were randomly selected by a pseudo-randomized number generator and invited by emails to participate in answering a web-based questionnaire. Over three months, 429 participants completed the survey, resulting in a more than 90% response rate. Sixty-eight dentists were excluded due to having a history of autoimmune disease, orthopedic trauma, or congenital disease that affect motor/hand function. The remaining 361 dentists were included in the study, fulfilling the minimum sample size requirements for both the first CMC joint osteoarthritis (174 subjects) and the Carpal Tunnel Syndrome (326 subjects). The sample size requirements were calculated based on different proportions from previous studies [23,24].

Questionnaire

The questionnaire contained 47 questions and took less than 5 minutes to be completed. In addition to the demographic data, work, medical, and past surgical history, we adapted two previously validated questionnaires; the Thumb Disability Exam (TDX) and the Boston Carpal Tunnel Syndrome Questionnaire (BCTQ) [25,26]. Any repeated question was only used once in our model. The final questionnaire was reviewed by 10 dentists who confirmed its relevance and validity.

The questionnaire contains three main parts as follows (see attached form):

Part I: Demographics and Health Information: This part is composed of 14 questions about age, gender, job title and status, years of practice, sub-specialty, weekly clinical working hours, hand dominance, pre-existing medical conditions, past surgical history, and previous diagnosis with first CMC joint osteoarthritis or CTS.

Part II: The Thumb Disability Exam (TDX), which composes 20 questions.

Part III: Boston Carpal Tunnel Syndrome Questionnaire (BCTQ) comprises 13 questions (after omitting the already existing questions in part II). The original BCTQ comprises two main parts: the symptom severity scale (SSS), which comprises 11 questions, and the functional status scale (FSS), which comprises eight questions.

Data management and analyses

The Statistical Package for Social Sciences (SPSS) V.27 was used for data management and statistical analysis. Descriptive analysis was performed to calculate the frequencies and percentages of the demographics and other categorical variables. Since the continuous numeric outcomes of TDX did not meet the assumption of normality, they were converted to binary data as No Disability (=20) or Disability were reported (>20). The ordinal outcomes for SSS and FSS dependent variables were also converted to binary data as Asymptomatic (=20) or Symptomatic (>20). Univariate logistic regression analysis was performed first to explore the potential predictors for symptomatic CMC osteoarthritis and Carpal Tunnel Syndrome, then adjusted with multivariate binary logistic regression analysis. The level of significance was set at a = 0.05 for all tests.

Ethical Considerations

This cross-sectional study did not involve any intervention that may cause harm to the participants. No participant was subjected to pressure to decide whether to participate in this survey. The ethical approval was obtained from the institutional review board of the King Faisal Specialist Hospital Research Center, Riyadh, Saudi Arabia. The Research Advisory Council (RAC) number is 2201135

## Results

Demographic and health information

In this cross-sectional study, 361 participants completed the survey and satisfied the inclusion/exclusion criteria, with 195 (54%) females. The 20-29-year-old had the highest percentage (45%, 163) of participants among the other age groups. Being a general practitioner (50%) practicing general dentistry (%55) with an experience of 1-5 years in dentistry (46%) were the most dominant characteristics of our participants. Most of these participants (93%) were right-handed. Table 1 shows a detailed description of the participants' demographics and health information. 

**Table 1:** Demographic and Health Characteristics of Respondents (N=361)

Variable	Category	n (%)
Gender	Male	166 (46)
	Female	195 (54)
Age group	20-29	163 (45)
	30-39	110 (30)
	40-49	57 (16)
	50-59	28 (8)
	60 or above	3 (1)
Job title	General Practitioner	180 (50)
	Resident	61 (17)
	Specialist/Consultant	120 (33)
Years in dentistry	1-5	168 (46)
	6-10	72 (20)
	11-15	46 (13)
	16-20	26 (7)
	More than 20 years	49 (14)
Subspecialty	General Dentistry	199 (55)
	Endodontics	21 (6)
	Oral and Maxillofacial Surgery	14 (4)
	Oral Medicine/ Oral Pathology/Oral Radiology	7 (2)
	Orthodontics	27 (7.5)
	Pediatric Dentistry	38 (10.5)
	Periodontics	34 (9)
	Prosthodontics	21 (6)
Years of practice as specialist	1-5	230 (64)
	6-10	44 (12)
	11-15	31(8)
	16-20	32 (9)
	More than 20 years	24 (7)
Clinical working hours per week	Less than 10 hours	78 (22)
	10-20 hours	63 (18)
	21-30 hours	70 (19)
	31-40 hours	83 (23)
	More than 40 hours	67 (18)
Dominant hand	Left-handed	24 (7)
	Right-handed	337 (93)
Pain in hand, base of the thumb or wrist	No	241 (67)
	Yes (Left hand)	20 (5)
	Yes (Right hand)	100 (28)
Symptom Severity Scale (SSS)	No	139 (39)
	Yes	222 (61)
Functional status scale (FSS)	No	150 (42)
	Yes	211 (58)
The Thumb Disability Exam (TDX)	No	118 (33)
	Yes	243 (67)

Thumb Disability Exam (TDX)

Univariate logistic regression analysis indicates that the female gender (OR 2.35; 95% CI 1.50-3.70) is more likely to report disability in TDX than males. Dentists in the 30-39 and 40-49-year-old age groups were less likely to report thumb disability. Additionally, dentists with 6 to 20 years of experience or subspecialties in maxillofacial surgery or orthodontics showed fewer thumb disabilities than others (Table 2).

In multivariate logistic regression analysis, the adjusted odds of thumb disability were two times higher in female dentists than in male dentists (aOR 2.21; 95% CI 1.31-3.56). Dentists aged 50 years or above showed nine times higher odds of having a thumb disability (aOR 9.63; 95% CI 1.05-88.47). In addition, a subspecialty of maxillofacial surgery has an 80% reduction in the odds of having a thumb disability. Other predictor variables showed no significant associations with multivariate regression (Table 2).

**Table 2:** Logistic Regression Analysis of the Predictors of the First Carpometacarpal (CMC) Joint Osteoarthritis using the Thumb Disability Exam (TDX) (N=361) * This group was used as a reference.

Category	Univariate analysis		Multivariate analysis	
	OR (CI 95%)	p-value	*Adjusted OR (CI 95%)	p-value
Gender				
Male*(reference)				
Female	2.35 (1.50-3.70)	<0.001	2.21 (1.31-3.56)	<0.001
Age group				
20-29*(reference)				
30-39	0.41 (0.24-0.68)	<0.001	1.01 (0.44-2.41)	0.94
40-49	0.48 (0.25-0.91)	0.02	4.14 (0.71-24.07)	0.11
50 or above	1.36 (0.52-3.54)	0.54	9.63 (1.05-88.47)	0.045
Job title				
General Practitioner*(reference)				
Resident	1.26 (0.64-2.48)	0.51	2.13 (0.76-5.93)	0.15
Specialist/Consultant	0.44 (0.27-0.72)	<0.001	0.92 (0.35-2.40)	0.86
Years in dentistry				
1-5*(reference)				
6-10	0.36 (0.20-0.64)	<0.001	0.51 (0.21-1.25)	0.14
11-15	0.39 (0.20-0.78)	0.01	0.36 (0.10-1.26)	0.11
16-20	0.35 (0.15-0.83)	0.02	0.14 (0.02-1.09)	0.06
More than 20 years	0.76 (0.37-1.55)	0.44	0.43 (0.01-1.50)	0.11
Subspecialty				
General Dentistry*(reference)				
Endodontics	0.49 (0.19-1.21)	0.12	0.67 (0.18-2.47)	0.54
Oral and Maxillofacial Surgery	0.20 (0.07-0.63)	0.01	0.20 (0.05-0.91)	0.04
Oral Medicine/ Oral Pathology/Oral Radiology	0.27 (0.06-1.26)	0.10	0.46 (0.08-2.77)	0.40
Orthodontics	0.39 (0.17-0.87)	0.02	0.41 (0.13-1.29)	0.13
Pediatric Dentistry	1.17 (0.52-2.63)	0.71	0.90 (0.28-2.91)	0.86
Periodontics	0.59 (0.27-1.25)	0.17	0.64 (0.21-1.97)	0.44
Prosthodontics	0.59 (0.23-1.50)	0.27	0.76 (0.22-2.67)	0.67
Years of practice as specialist				
1-5*(reference)				
6-10	0.34 (0.18-0.66)	<0.001	0.81 (0.33-2.01)	0.65
11-15	0.86 (0.39-1.93)	0.72	2.13 (0.63-7.23)	0.23
More than 15	0.94 (0.50-1.78)	0.86	1.66 (0.39-7.09)	0.50
Clinical working hours per week				
Less than 10 hours*(reference)				
10-20 hours	0.60 (0.29-1.22)	0.16		
21-30 hours	0.66 (0.33-1.34)	0.25		
31-40 hours	0.81 (0.41-1.60)	0.54		
More than 40 hours	0.75 (0.37-1.54)	0.44		
Dominant hand				
Left-handed*(reference)				
Right-handed	1.03 (0.43-2.48)	0.94		

Boston Carpal Tunnel Syndrome Questionnaire (BCTQ)

Symptom Severity Scale (SSS)

Univariate logistic regression analysis indicates that the female gender (OR 1.5; 95% CI 1.002-2.350) is more likely to be symptomatic of CTS than the male gender. Dentists who have a job title of consultant/specialist, have been practicing dentistry for 6-10 years, or working 10-20 hours per week were less likely to report symptoms of CTS. In addition, right-handed dentists were two times more likely to have symptoms of CTS (Table 3).

After multivariate regression analysis, the only predictor variables that showed a significant association was gender, job title, and clinical working hours per week. The adjusted odds of SSS were 1.62 times higher in female dentists than in male dentists (aOR 1.62; 95% CI 1.62-2.58). Being a consultant dentist or working 10-20 hours per week reduces the odds of having symptoms of CTS in SSS (Table 3).

**Table 3:** Logistic Regression Analysis of the Predictors of Carpal Tunnel Syndrome (CTS) Symptom Severity Scale (SSS) (N=361) * This group was used as a reference.

Category	Univariate analysis		Multivariate analysis	
	OR (CI 95%)	p-value	*Adjusted OR (CI 95%)	p-value
Gender				
Male* (reference)				
Female	1.5 (1.002-2.350)	0.049	1.62 (1.62-2.58)	0.043
Age group				
20-29* (reference)				
30-39	0.692 (0.42-1.13)	0.14		
40-49	1.17 (0.63-2.26)	0.58		
50 or above	1.72 (0.72-4.08)	0.22		
Job title				
General Practitioner*(reference)				
Resident	0.72(0.40-1.38)	0.28	0.93 (0.48-1.81)	0.83
Specialist/Consultant	0.61(0.38-0.98)	0.042	0.52 (0.28-0.98)	0.043
Years in dentistry				
1-5*(reference)				
6-10	0.55 (0.32-0.97)	0.038	0.75 (0.36-1.54)	0.43
11-15	1.10 (0.55-2.17)	0.79	1.30 (0.50-3.42)	0.59
16-20	0.94 (0.40-2.19)	0.88	1.39 (0.33-5.87)	0.66
More than 20 years	1.46 (0.73-2.93)	0.28	1.95 (0.39-9.85)	0.42
Subspecialty				
General Dentistry*(reference)				
Endodontics	0.55 (022-1.35)	0.19		
Oral and Maxillofacial Surgery	0.66 (0.22-1.99)	0.46		
Oral Medicine/ Oral Pathology/Oral Radiology	1.24 (0.23-6.57)	0.80		
Orthodontics	0.46 (0.21-1.04)	0.06		
Pediatric Dentistry	0.95 (0.46-1.99)	0.90		
Periodontics	0.50 (0.24-1.03)	0.06		
Prosthodontics	0.45 (0.18-1.17)	0.09		
Years of practice as specialist				
1-5*(reference)				
6-10	0.61 (032-1.16)	0.13		
11-15	1.63 (0.72-3.70)	0.24		
More than 15	1.82 (0.95-3.48)	0.07		
Clinical working hours per week				
Less than 10 hours*(reference)				
10-20 hours	0.40 (0.20-0.79)	0.01	0.44 (0.21-0.90)	0.024
21-30 hours	0.79 (0.41-1.55)	0.50	0.84 (0.41-1.71)	0.62
31-40 hours	1.10 (0.57-2.11)	0.78	1.11 (0.55-2.23)	0.78
More than 40 hours	1.16 (0.58-2.33)	0.68	1.08 (0.51-2.30)	0.84
Dominant hand				
Left-handed*(reference)				
Right-handed	2.37 (1.02-5.51)	0.04	2.31 (0.95-5.62)	0.07

Functional Status Scale (FSS)

Most of the predictor variables showed at least one significant association among their groups in the univariate logistic regression analysis of FSS. However, only gender and dental subspecialty variables revealed a significant association in multivariate logistic regression analysis (Table 4). Female dentists were two times more likely to report hand performance difficulties than male dentists (aOR 2.21; 95% CI 1.36-3.57). In addition, endodontists showed an 85% reduction in the likelihood of reporting hand disability in FSS of BCTQ (aOR 0.15; 95% CI 0.04-0.58) (Table 4).

**Table 4:** Logistic Regression Analysis of the Predictors of Carpal Tunnel Syndrome (CTS) Functional Status Scale (FSS) (N=361) * This group was used as a reference.

Category	Univariate analysis		Multivariate analysis	
	OR (CI 95%)	p-value	*Adjusted OR (CI 95%)	p-value
Gender				
Male*(reference)				
Female	2.66 (1.73-4.10)	<0.001	2.21 (1.36-3.57)	<0.001
Age group				
20-29*(reference)				
30-39	0.622 (0.38-1.02)	0.06	1.23 (0.52-2.89)	0.64
40-49	0.39 (0.21-0.72)	<0.001	2.07 (0.40-10.79)	0.39
50 or above	1.31 (0.57-3.04)	0.52	4.33 (0.53-35.15)	0.17
Job title				
General Practitioner* (reference)				
Resident	0.91(0.50-1.66)	0.76	1.46 (0.57-3.74)	0.43
Specialist/Consultant	0.50(0.31-0.80)	<0.001	1.20 (0.48-3.02)	0.70
Years in dentistry				
1-5*(reference)				
6-10	0.45 (0.26-0.79)	<0.001	0.49 (0.20-1.17)	0.11
11-15	0.52 (0.27-1.00)	0.05	0.36 (0.10-1.29)	0.12
16-20	0.21 (0.09-0.51)	<0.001	0.14 (0.2-1.06)	0.06
More than 20 years	0.75 (0.39-1.45)	0.39	0.30 (0.03-2.89)	0.30
Subspecialty				
General Dentistry*(reference)				
Endodontics	0.13 (0.04-0.39)	<0.001	0.15 (0.04-0.58)	0.01
Oral and Maxillofacial Surgery	0.22 (0.07-0.72)	0.01	0.27 (0.06-1.24)	0.09
Oral Medicine/ Oral Pathology/Oral Radiology	0.22 (0.04-1.15)	0.07	0.34 (0.05-2.42)	0.28
Orthodontics	0.58 (0.26-1.31)	0.19	0.62 (0.21-1.85)	0.39
Pediatric Dentistry	1.04 (0.50-2.17)	0.91	0.98 (0.33-2.91)	0.97
Periodontics	0.78 (0.37-1.63)	0.50	0.92 (0.31-2.76)	0.89
Prosthodontics	0.88 (0.35-2.23)	0.79	1.12 (0.32-3.91)	0.86
Years of practice as specialist				
1-5*(reference)				
6-10	0.33 (0.17-0.63)	<0.001	0.79 (0.31-1.99)	0.61
11-15	0.80 (0.37-1.71)	0.56	2.65 (0.76-9.30)	0.13
More than 15	0.71 (0.40-1.29)	0.26	1.22 (0.28-5.30)	0.79
Clinical working hours per week				
Less than 10 hours*(reference)				
10-20 hours	0.49 (0.25-0.98)	0.04	0.63 (0.28-1.41)	0.26
21-30 hours	0.56 (0.28-1.10)	0.09	0.61 (0.28-1.34)	0.21
31-40 hours	0.43 (0.22-0.82)	0.01	0.52 (0.25-1.11)	0.09
More than 40 hours	0.62 (0.31-1.23)	0.17	0.82 (0.37-1.84)	0.63
Dominant hand				
Left-handed*(reference)				
Right-handed	1.21 (0.53-2.77)	0.66		

## Discussion

Musculoskeletal disorders are common among dentists and are considered a major occupational concern [7]. Healthcare workers in the dental field are at increased risk of developing upper extremity symptoms, CTS, and back pain [27]. Multiple factors can explain the increased prevalence of cumulative musculoskeletal trauma disorders among dentists: the frequent use of force, vibrating tools, faulty postures, lengthy working hours, and an increased BMI of the dentist [27-31]. Furthermore, wrist-related pain and neuropathy were frequently reported among dental practitioners worldwide [7,19,32-34].

In this study, maxillofacial surgeons reported the lowest rate of thumb disability complaints. In support of our findings, the lowest reporting rate of WMSD and CTS was among maxillofacial surgeons and orthodontists in a study conducted among dentists in Jeddah, Saudi Arabia [11]. In contrast, another study found that orthodontists had the highest prevalence of wrist symptoms among dentists [32]. Furthermore, according to Alghadir et al., pediatric dentists are at the highest risk for MSDs. Most of the MSDs regions identified by pediatric dentists in the latter study are related to the knees, explaining the discrepancy with the current study results [35].

In our study, dentists aged 50 years or above showed nine times higher odds of having a thumb disability. This could be attributed to multiple factors, such as repetitive trauma due to multiple years of working in unfavorable conditions. Thus, an advanced stage of damage to the joint. In a study conducted in Madhya Pradesh, India, to assess work-related musculoskeletal disorders (WMSDs) among dental practitioners, wrist pain was reported by 17.89% of the participants. There was a significant association between musculoskeletal pain and dentists who did not have a proper fitness regimen and those who worked in suboptimal situations, such as working without an assistant, standing, or using direct vision [19]. Additionally, another study found that the symptoms of MSDs, including finger pain, were directly proportional to the number of years of practice [36].

In this study, right-handed dentists reported more symptoms when compared to left-handed ones. This is supported by a study conducted in Slovenia to measure MSDs among dental workers, where 23.8% of the responders complained of right wrist pain [33]. It could be attributed to the number of right-handed responders to our study.

Additionally, in this study, females were more likely to report disability in TDX than males. WMSDs had an increased incidence and prevalence among females in previous studies. They were attributed to having psychosocial stress and women who work in similar jobs to dentistry, such as the service industry [37-39]. In contrast, a systematic review among dentists in Iran found no gender difference [34]. In the third part of the survey, we used the Boston Carpal Tunnel Syndrome Questionnaire (BCTQ) to measure the symptoms of CTS. Symptoms of CTS were reported frequently in different professions, such as food processors, assembly workers, drillers, and dental hygienists [40-42]. However, the prevalence among dentists was more when compared to the general population [43-47].

In the present study, specialists/consultants had the lowest reporting rate of CTS symptoms. This can be attributed to gaining experience in posture and fewer working hours as the practitioner gets promoted to a consultant/specialist. This is supported by another study in Malaysia, which revealed that dental practitioners would be less likely to develop symptoms of carpal tunnel syndrome with more years of practice [46].

Additionally, another study in Pakistan supported the finding that up to 20 years of practice, dentists will usually report more symptoms of CTS [48]. However, multivariate analysis revealed no increased risk with increasing years of practice. Additionally, our study showed that the number of hours the dentist spend working is directly proportional to the presence of CTS symptoms and hand/finger pain. Previous studies conducted among Saudi and Iranian dentists have shown similar results [44,23,49].

Further to the previous finding, females were more likely to be symptomatic of CTS than males. Multiple previous studies had findings that were consistent with this one [42,23,50]. Females' risk factors could be attributed to hormonal changes, hormonal therapy, pregnancy, and sensitivity to reporting symptoms [51-53]. In contrast, another study conducted by McDiarmid et al. found no gender difference [54].

Moreover, the statistical analysis has shown that right-handed dentists were two times more likely to have symptoms. However, the percentage of right-handed people who participated in the study was 93% which means that the dominant hand will more likely be affected. This finding is consistent with many other studies where the dominant hand tends to be affected more by CTS than the other hand [23,49]. Interestingly, endodontists showed an 85% reduction in the likelihood of reporting hand disability in FSS of BCTQ. Contrary to other studies that suggest pediatric dentists have the highest percentage of CTS symptom reporting, our data showed a high but not statistically significant rate of CTS symptom and disability reporting [23].

Our results suggest that dentists are at an increased risk of developing CTS and first CMC joint osteoarthritis. It is vital to educate young dentists about hand/wrist ergonomics in specific and the whole body in general, as it plays a vital role in prevention [32,23]. The major strengths of this study were the response rate (more than 90%) and having the usual sample size. One limitation is the self-reporting nature of the symptoms in the survey, which could have been affected by external bias.

This is the first research to study the prevalence of both CTS and the first CMC joint osteoarthritis among dentists in the whole Kingdom of Saudi Arabia. Additional research is encouraged among larger groups to assess further the relationship between psychosocial factors and the perception of general health as demanding jobs without breaks may result in an increasing need for recovery and worst appreciated general health among certain professions compared to others [32,55].

## Conclusions

This study has perceived that the prevalence of thumb disability was present with a significant p-value among females aged above 50 years old and with lower levels of experience in dentistry. In contrast, maxillofacial surgeons have less risk of thumb disability. Regarding CTS, female gender, lower level of experience, and higher working hours showed significantly increased risk.
